# Supplementary material for: In Vitro Evaluation of the Safety and Efficacy of Cibisatamab Using Adult Stem Cell-Derived Organoids and Colorectal Cancer Spheroids
Source: Cancers (Basel). 2025 Jan 17;17(2):291. doi: 10.3390/cancers17020291 (PMC11763756; doi:10.3390/cancers17020291)
Supplement: Supplementary file 1 [file cancers-17-00291-s001.zip › cancers-3371752-supplementary.pdf]

Supplemental Information

Supplemental Table S1. Two-way ANOVA with multiple comparisons test was performed and the mean of each column compared to the mean of the reference column (10 μM MKN-45). ****P < 0.0001. Only significant values are displayed (P <0.05).

Read-out	Cell line	DLD-1					
	Treatment dose	0.001 μM	0.01 μM	0.1 μM	1 μM	10 μM	100 μM
Ethidium	MKN-45 (10 μM)	****	****	****	****	****	****
ATP		****	****	****	****	****	****
LDH		****	****	****	****	****	****

Supplemental Table S2. Two-way ANOVA with multiple comparisons test was performed and the mean of each column compared to the mean of the reference column (10 μM Rectum). **P < 0.01, and ****P < 0.0001. Only significant values are displayed (P <0.05); ns (not significant).

Read-out	Organoid line	Small intestinal organoid					
	Treatment dose	0.001 μM	0.01 μM	0.1 μM	1 μM	10 μM	100 μM
Ethidium	Rectum organoid (10 μM)	****	****	****	****	****	****
ATP		ns	ns	ns	**	**	**

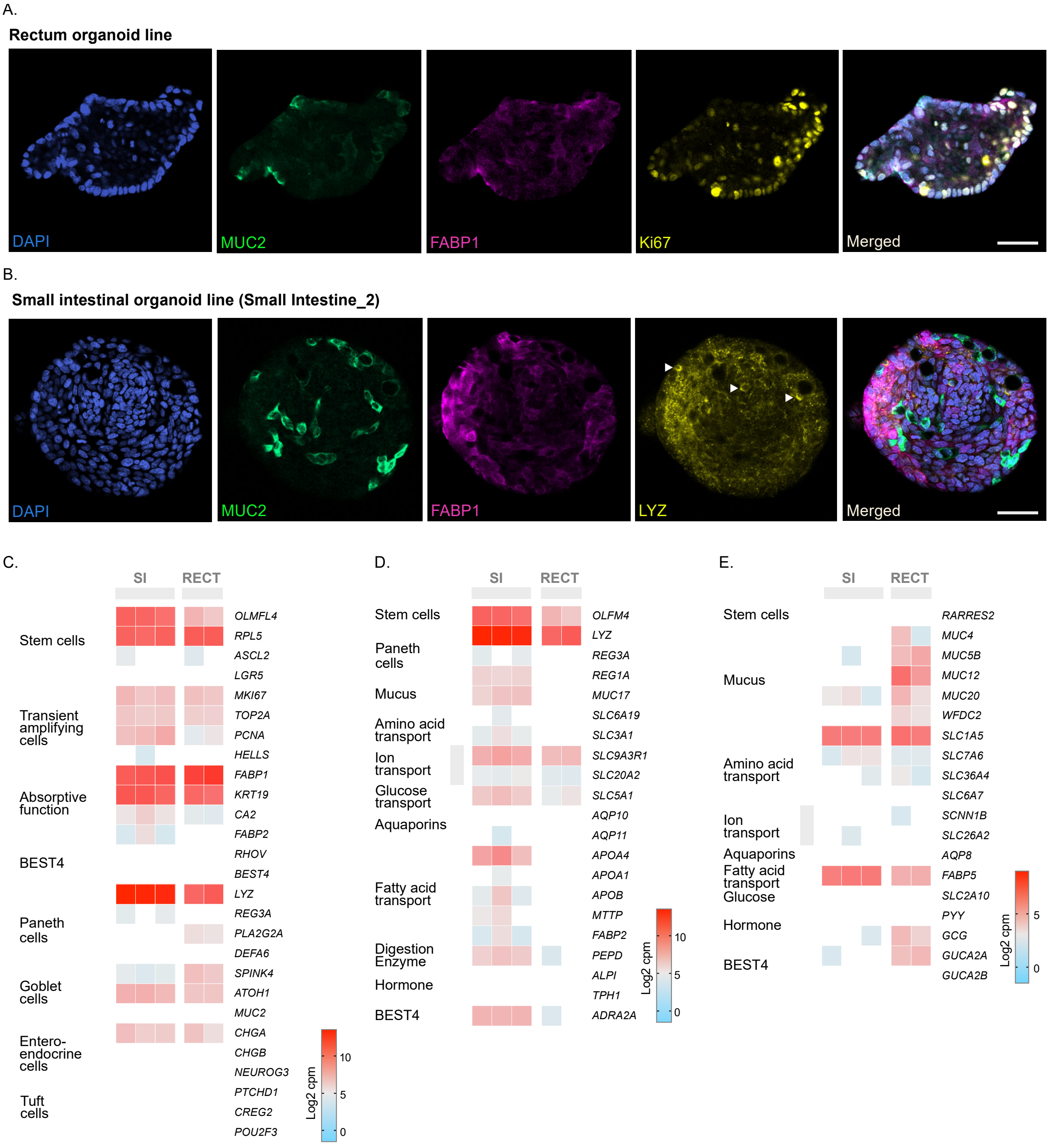

Supplemental Figure S1. Characterization of rectal and small intestinal (Small intestine_2) organoid lines used in the study.
(A) Phenotypic characterization of rectal organoid line through Immunofluorescent staining. Rectal organoids show expression of MUC2 (Goblet cells), FABP1 (Enterocytes; absorptive cells), and Ki67 (proliferative cells). **(B)** Phenotypic characterization of small intestinal (SI) organoid line through Immunofluorescent staining. SI organoids show expression of MUC2 (Goblet cells), FABP1 (Enterocytes; absorptive cells), and Lysozyme (secreted by Paneth cells; white arrowheads). Scale bar 50µm. (**C–E)** Bulk-RNA-based characterization of organoid lines used in this study. Three biological replicates of the small intestinal line (Small intestine_2) and two biological replicates of the rectal organoid line were sequenced and canonical pathways (**C**), small intestinal hallmarks (**D**) and colon/rectal hallmarks (**E**) were analyzed. Shown as log2 count per million (cpm).

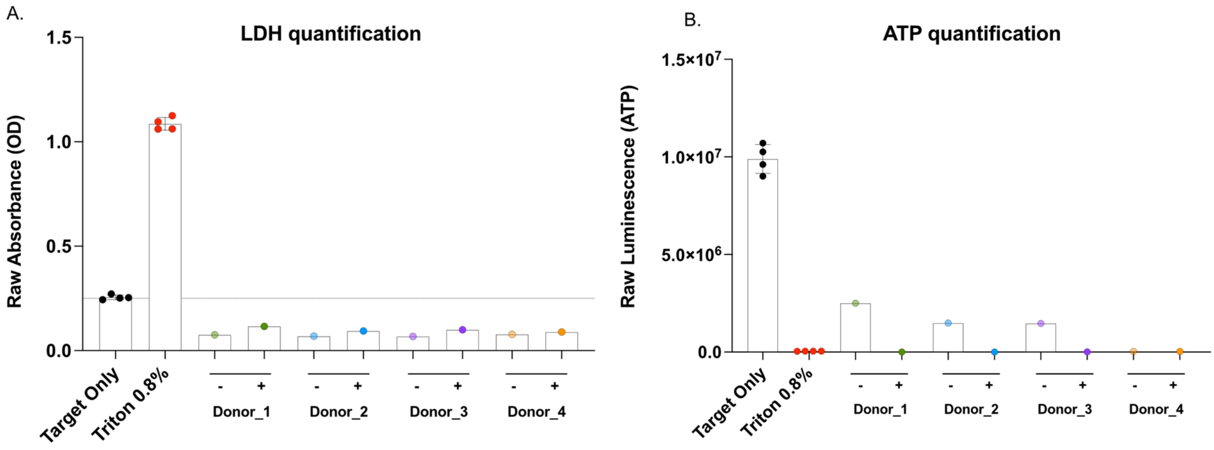

Supplemental Figure S2. Validation of read-out-specificity by single culture of human hPBMCs of 4 different donors.

(A,B) hPBMCs treated over-night with 0.8% Triton-X (+) release low levels of LDH and show low levels of ATP compared to target cells. These results confirm the read-out specificity to detect target cell death in our RDL assays. The wells were seeded with the highest concentration of hPBMCs (E:T, 10:1). Data represent mean ± SD from 3 different hPBMCs donors tested in duplicates.

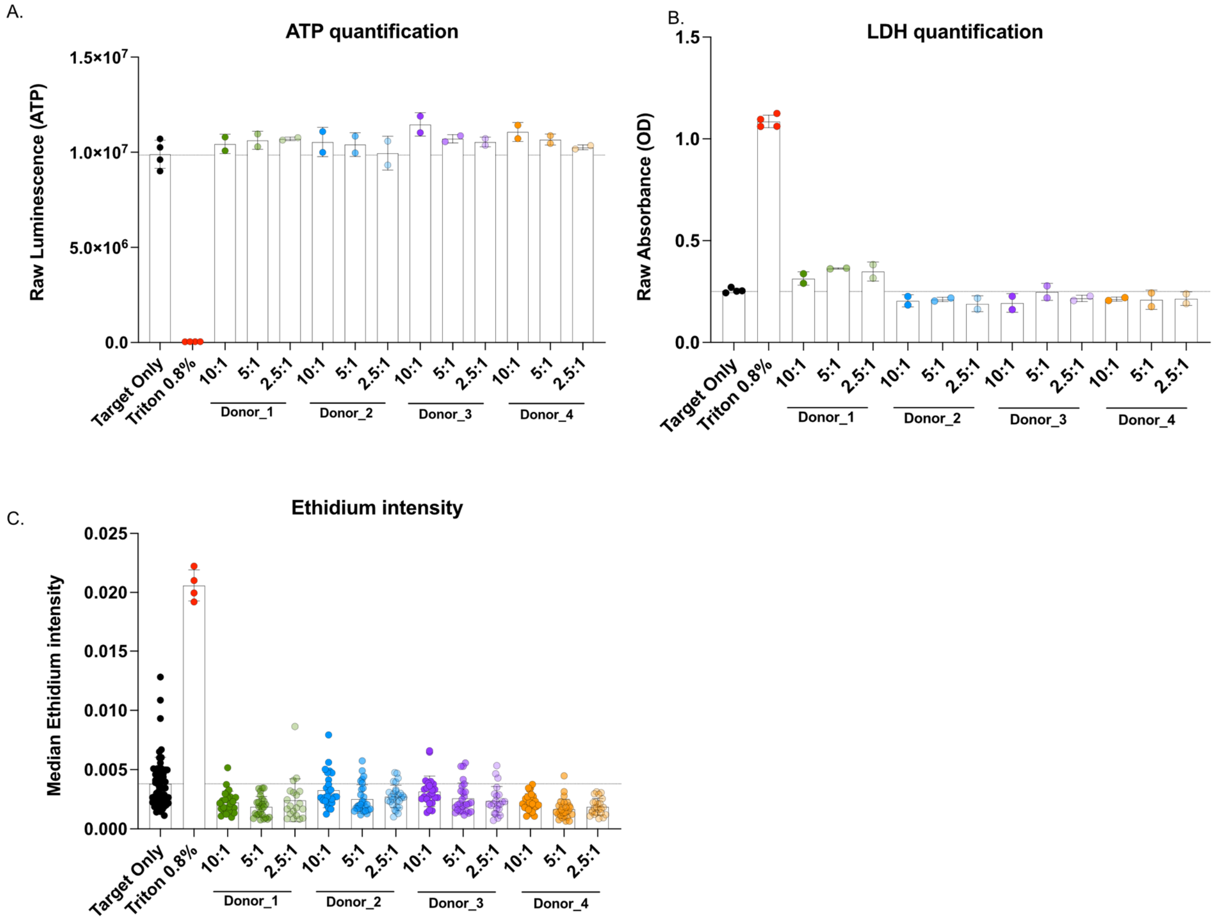

Supplemental Figure S3. Validation of read-out-specificity by single culture of human hPBMCs of 4 different donors.

(A–C) To evaluate the potential spontaneous killing induced by hPBMCs on target cells, hPBMCs were co-cultured with targets cells in the absence of any treatment and ATP content **(A)**, LDH release **(B)** and median ethidium intensity **(C)** were quantified.

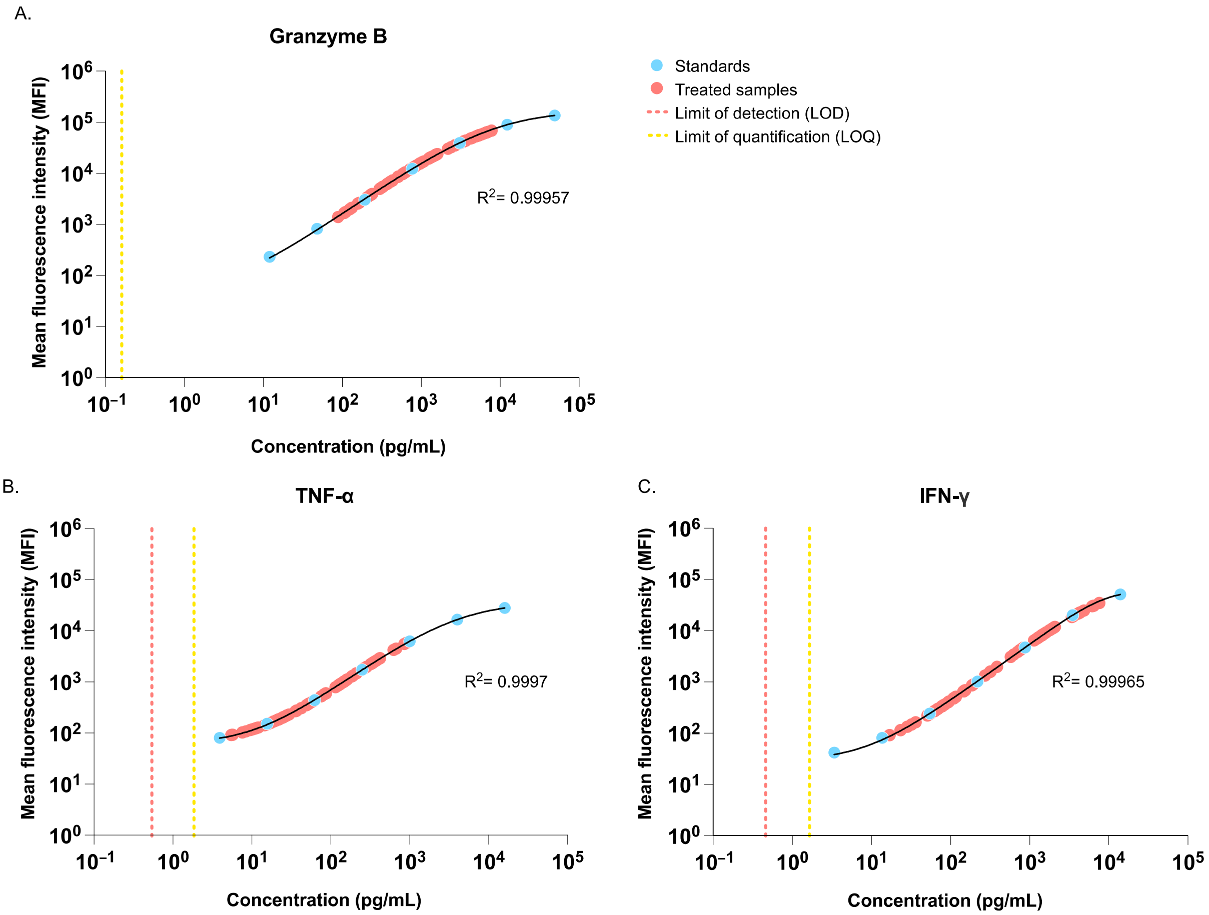

Supplemental Figure S4. Standard curves and 5PL fitting parameters of Granzyme B, TNF-α and INF-γ. Standard curves for (**A**) Granzyme B, (**B**) TNF-α and (**C**) INF-γ exhibited a R^2 value > 99%. Data represents duplicates of each standard dose; 8 different doses have been measured as recommended by the manufacturer (dose 8 = blank).

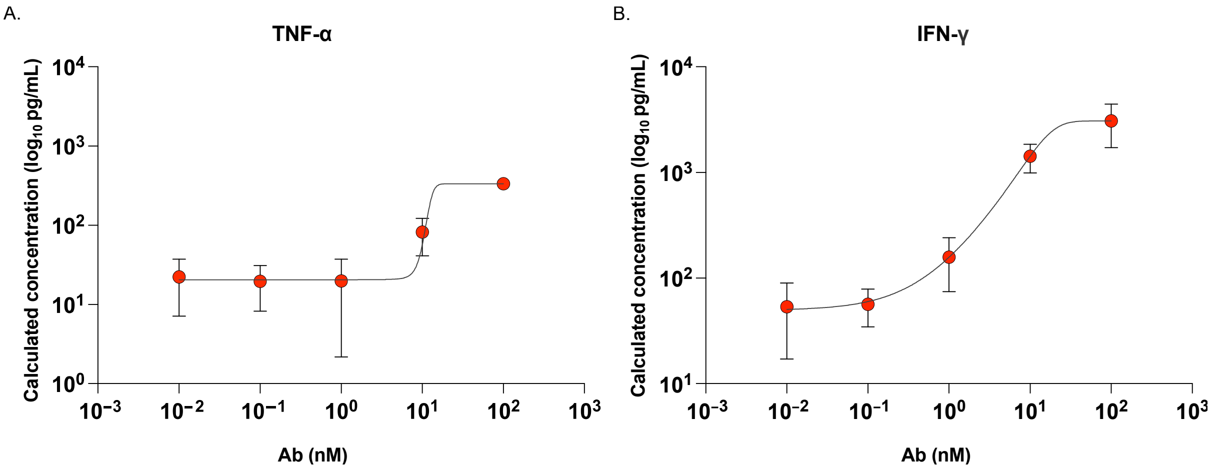

Supplemental Figure S5. TNF-α and INF-γ quantification after 72h co-culture of 3D grown MKN-45 spheroids in the presence of hPBMCs treated at different doses of CEA TCB.

A. Quantification of TNF-α, and B. Quantification of INF-γ released in the supernatant after 72h RDL assay by flow cytometry. Data represent nonlinear regression analysis of the mean ± SD from 3 different hPBMCs donors tested in duplicates. Ordinary 1-way ANOVA with Dunnett's multiple comparisons test was performed and the mean of each column compared to the mean of MKN-45 treated at 100 nM.
